# Adult onset egg allergy: a case report

**DOI:** 10.1186/s12948-021-00156-7

**Published:** 2021-10-04

**Authors:** Elisa Maria Cremonte, Eugenia Galdi, Chiara Roncallo, Elisa Boni, Luigi Giovanni Cremonte

**Affiliations:** 1 Internal Medicine Resident, University of Pavia, Pavia, Italy; 2 SS of Allergology ASL AL, Novi Lingure, AL Italy; 3 Laboratorio Unico Metropolitano, Maggiore Hospital AUSL Bologna, Largo Nigrisoli n. 2, 40133 Bologna, Italy

**Keywords:** Egg allergy, Food allergy, Ovomucoid, Ovalbumin

## Abstract

**Background:**

Egg allergy is one of the most frequent food allergies in childhood while adult onset of egg allergy is a rare condition.

**Case presentation:**

We report the case of a 30 years old man sent to our center in order to investigate gastrointestinal symptoms occurring since 2 years after egg and derivatives intake. He did not suffer from egg or other food allergies in childhood. He is an active smoker with a contact dermatitis related to nickel and mild allergic rhinoconjunctivitis to grass pollen. Skin prick test and serum specific IgE to egg were performed and revealed sensitization to egg proteins.

**Conclusions:**

Even though IgE-mediated egg allergy affects children, this report witnesses a rare case of adult onset.

## Introduction

Although egg allergy prevails in pediatric population, some studies have described the persistence or newly onset egg sensitization through adults [1]. A failure in oral tolerance or a breakdown in previously acquired tolerance results in food hypersensitivity. Egg allergy is frequently observed during the first years of life, according to its alimentary introduction. There is a possible resolution of egg allergy within 10–15 years from the diagnosis. Symptoms include early onset of IgE-mediated urticaria, eczema, abdominal pain and vomiting. In some more severe cases anaphylaxis with dyspnea and hypotension can occur. Non-IgE mediated symptoms include eosinophilic diseases of the gut or egg-induced enterocolitis [2, 3]. Consumption of baked egg by raw egg allergic children does not seem to either induce or accelerate the development of tolerance in this population [4]. There is evidence of a strong association between sensitization to egg during infancy and, later, to inhalant allergens.

The rare adult-onset egg allergy is often associated with previous personal history of atopy or other food intolerances [5–7]. Some studies have shown that stress conditions or alteration of intestinal microbiota could be responsible for the loss of tolerance toward some food antigens [1]. Intestinal inflammatory disorders, such as Crohn disease, celiac disease, or ulcerative colitis favor the development of food allergy by altering intestinal permeability. It has been reported a case of late-onset egg allergy following the diagnosis of Hodgkin’s lymphoma and start of chemotherapy and the authors speculated an alteration involving bowel mucous membrane [8].

Diagnosis of egg allergy includes anamnesis, skin prick tests and serum specific IgEs [9]. The most significant antigenic components of egg white are ovomucoid (Gal d 1—thermo stable), ovalbumin (Gal d 2—thermolabile), ovotransferrin (Gal d 3), and lysozime (Gal d 4). Depending on the sensitization pattern, patients with egg allergy can manifest symptoms with cooked or raw egg. In some patients a relationship between hypersensitivity secondary to bird antigens exposure and allergy to egg yolk has been documented [1]. This is known as bird-egg syndrome [10]. It derives from the sensitization to the chicken serum protein called alpha livetin (Gal d 5), that is also represented in egg yolk. It is characterized by respiratory and gastrointestinal symptoms after egg intake or after exposure to feathers and droppings of birds. Allergy to other aeroallergens is frequently documented in individuals with bird-egg syndrome.

Currently, there is active research on trying oral immunotherapy to desensitize people to egg allergens [11–13].

## Case report

We report a rare case of adult-onset egg allergy. A 30 years old man referred to our center in order to investigate gastrointestinal symptoms (vomit, abdominal pain and diarrhea) that have been occurring for 2 years. He reports that symptoms occur within few minutes after egg or egg derivatives ingestion: pasta carbonara, mayonnaise, fried egg and meringue. Conversely, he tolerates baked egg (at cooking temperature greater than 160 °C for 25–30 min) independently from a flour matrix presence.

No familial history for atopy is present. In childhood, he did not suffer from atopic dermatitis nor egg or other food allergies while a history of contact dermatitis related to nickel and mild allergic rhinoconjunctivitis to grass pollen is reported. He is not affected by immunodeficiency or chronic inflammatory bowel disease, nor disorders affecting the biliary tract or intolerance to lactose. He is an active smoker. He does not have any occupational exposure risk.

## Materials and methods

Skin prick test with a large panel of commercial food and common inhalant allergen extracts (Lofarma, Milan, Italy) and serum specific IgEs (Thermo Fisher Scientific, US) to egg extract and to ovomucoid, ovalbumine and lysozyme were performed. According to our laboratory ranges, values ≥ 0.10 kUa/l are considered positive.

Prick by prick test with white and yolk from raw egg and cooked egg was done. The egg was boiled at 100 °C for ten minutes and yolk was mechanically separated from white.

## Results

The prick test with commercial extracts showed a positive response to egg white and grasses, while animal epithelia, dust mites and molds were negative.

Prick by prick test was positive for raw and boiled egg white (diameter of wheals were 15 mm × 8 mm and 4 mm × 2 mm respectively).

Serum specific IgEs were positive for egg white (3.52 kUa/l), showing a strong positivity for Gal d 2-ovalbumin (8.87 kUa/l) and low positive value for Gal d 1-ovomucoid (0.10 kUa/l) according to laboratory ranges (positive result ≥ 0.10 kUa/l).

Diagnosis of egg allergy was confirmed. The patient was advised to avoid ingestion of egg and egg derivatives. Self-injectable epinephrine and adequate pharmacotherapy in the event of accidental ingestion was delivered.

## Discussion

Adult onset egg allergy has been rarely reported in literature and, generally, it is preceded by a certain degree of egg hypersensitivity in childhood [14]. Our case is noteworthy and peculiar because symptoms occurred only in adulthood and history of occupational or home exposure to allergens was absent. Moreover, in our patient the clinical presentation of IgE mediated egg allergy is atypical since other extra-gastrointestinal symptoms are not present but clinical correlation between strong ovalbumin sensitization and occurrence of symptoms with raw egg or with cooked egg at low temperature is strong. The patient did not complain about respiratory symptoms and skin prick test were negative for animal fur, so bird-egg syndrome was excluded.

We may speculate that the clinical presentation of egg allergy, characterized by gastrointestinal disorders in the absence of any systemic symptom, is dependent from the presence of an intact intestinal mucosa.

## Data Availability

Not applicable.
